# Human Motor Cortex Functional Changes in Acute Stroke: Gender Effects

**DOI:** 10.3389/fnins.2016.00010

**Published:** 2016-01-29

**Authors:** Vincenzo Di Lazzaro, Giovanni Pellegrino, Giovanni Di Pino, Federico Ranieri, Fiorenza Lotti, Lucia Florio, Fioravante Capone

**Affiliations:** ^1^Unit of Neurology, Neurophysiology, Neurobiology, Department of Medicine, Università Campus Bio-Medico di RomaRome, Italy; ^2^Fondazione Alberto Sordi – Research Institute for AgeingRome, Italy; ^3^Multimodal Functional Imaging Laboratory, Montreal Neurological Institute – McGill UniversityMontreal, QC, Canada; ^4^Orthopaedic and Trauma Surgery Unit, Università Campus Bio-Medico di RomaRome, Italy

**Keywords:** acute cerebral infarction, gender, neurophysiology, stroke, transcranial magnetic stimulation

## Abstract

The acute phase of stroke is accompanied by functional changes in the activity and interplay of both hemispheres. In healthy subjects, gender is known to impact the functional brain organization. We investigated whether gender influences also acute stroke functional changes. In thirty-five ischemic stroke patients, we evaluated the excitability of the affected (AH) and unaffected hemisphere (UH) by measuring resting and active motor threshold (AMT) and motor-evoked potential amplitude under baseline conditions and after intermittent theta burst stimulation (iTBS) of AH. We also computed an index of the excitability balance between the hemispheres, laterality indexes (LI), to evidence hemispheric asymmetry. AMT differed significantly between AH and UH only in the male group (*p* = 0.004), not in females (*p* > 0.200), and both LI_AMT_ and LI_RMT_ were significantly higher in males than in females (respectively *p* = 0.033 and *p* = 0.042). LTP-like activity induced by iTBS in AH was more frequent in females. Gender influences the functional excitability changes that take place after human stroke and the level of LTP that can be induced by repetitive stimulation. This knowledge is of high value in the attempt of individualizing to different genders any non-invasive stimulation strategy designed to foster stroke recovery.

## Introduction

Gender related functional asymmetries between the two cerebral hemispheres have been documented in healthy human brain (Tomasi and Volkow, 2012). It has been suggested that they might be responsible for gender differences in cognitive styles (Proust-Lima et al., 2008), in the incidence of neuropsychiatric disorders (Narr et al., 2001; Baron-Cohen et al., 2005), and for a gender-specific influence on the functional outcome after unilateral cerebral lesion (Draca, 2010). Sex-related differences have been also reported after stroke with a worse functional outcome in women (Lisabeth et al., 2015), however the causes of this sex disparity in stroke outcome are still largely unknown because demographics, prestroke and clinical factors cannot explain it. One possibility is that gender has a significant influence on the functional changes underlying recovery that take place in the brain after a stroke. Non-invasive brain stimulation techniques provide the opportunity for the functional evaluation of the human brain. Thanks to these techniques it has been shown that pronounced asymmetrical functional changes take place in cortex in the acute phase of stroke (Di Pino et al., 2014a,b). These changes involve both the affected (AH) and unaffected (UH) hemispheres and might be correlated with long term recovery (for review see Di Pino et al., 2014a). Along this line, it is still unknown whether gender has an effect in stroke-related acute functional changes in the excitability of AH and UH.

The present study aims at investigating whether gender influences the cortical functional changes observed in the acute phase of stroke. To this end, in patients with acute stroke we evaluated motor cortex excitability by using single pulse transcranial magnetic stimulation (TMS) and the propensity of the cortex to undergo LTP- and LTD-like plasticity by means of a repetitive TMS (rTMS) paradigm, known as intermittent theta burst stimulation (iTBS). iTBS produces LTP-like changes in the stimulated hemisphere and LTD-like changes in the contralateral hemisphere (Di Lazzaro et al., 2008; Suppa et al., 2008). Similar interhemispheric effects have been observed also using a different TBS protocol known as continuous TBS, a rTMS paradigm that produces opposite effects on cortical excitability with LTD-like changes in the stimulated hemisphere and LTP-like changes in the contralateral hemisphere (Stefan et al., 2008).

Electrophysiological findings after single pulse TMS and after iTBS were compared between genders.

## Materials and methods

### Patients

Thirty-five patients with first-ever stroke were recruited (mean age = 71.4 years, SER = 1.96, 15F). Inclusion criteria were: (1) single ischemic stroke (both cortical and subcortical) involving the middle cerebral artery territory; (2) less than 10 days post-stroke; (3) hand weakness; (4) recordable muscle evoked potential (MEP) after TMS of the AH. Exclusion criteria were: (1) history of seizure; (2) hemorrhagic stroke; (3) concomitant neurological or other severe medical problems; (4) complete paralysis of the hand; (5) inability to give informed consent; (6) concomitant treatment with drugs acting on the central nervous system; (7) contraindications for TMS studies. In order to identify at risk patients for post-stroke epilepsy, all patients underwent an EEG before entering the study and none of them showed any epileptic abnormality (Rossini and Johnston, 2005). The evaluation of neurological impairment was based on the National Institutes of Health Stroke Scale (NIHSS).

All patients underwent brain MRI with a 1.5-T scanner (GE Signa; General Electric, Milwaukee, WI), and lesion size was estimated by using the Alberta Stroke Program Early CT Score (ASPECTS) (Barber et al., 2000).

All the patients signed a written informed consent form. This study was conducted in accordance with the Helsinki Declaration of 1975 and was approved by the Ethics Committee of Campus Bio-Medico University of Rome.

### Magnetic stimulation

#### Motor cortex excitability to single pulse TMS

Magnetic stimulation was performed with a high-power Magstim 200 (MagstimCo., Whitland, Dyfed). A figure-of-eight coil with external loop diameters of 9 cm was held over the motor cortex at the optimum scalp position to elicit MEPs in the contralateral first dorsal interosseous muscle (FDI). The induced current flowed in a postero-anterior direction. We evaluated the threshold and amplitude of MEPs. The resting motor threshold (RMT) was defined as the minimum stimulus intensity, expressed as the percentage of the maximal output intensity deliverable by the stimulator, which produced a liminal MEP (about 50 μV in 50% of 10 trials) at rest (Rossini, 2014). The active motor threshold (AMT) was defined as the minimum stimulus intensity that produced a liminal MEP (about 200 μV in 50% of 10 trials) during isometric contraction of the tested muscle (Rossini, 2014). We evaluated the RMT, AMT, and MEP amplitude elicited stimulating both the AH and UH. The MEP amplitude was evaluated using a stimulus intensity of 120% RMT with the muscle at rest. Audio-visual feedback of the electromyographic (EMG) signal at high gain was given to subjects in order to assist them in maintaining complete relaxation; trials contaminated by EMG activity were discarded. Ten data sweeps were collected, and the mean peak-to-peak amplitude of the MEPs was calculated.

#### Intermittent theta burst stimulation

iTBS was delivered over the affected motor cortex “hot spot” for MEPs in the contralateral FDI muscle using a MagPro stimulator (Medtronic A/S Denmark) connected to a figure-of-eight coil (MCF B65). The magnetic stimulus had a biphasic waveform with a pulse width of about 280 μs and a maximum magnetic field strength of 1.5 T. The initial direction of the current induced in the brain was anterior to posterior. The stimulation intensity was defined in relation to the AMT evaluated using the MagPro stimulator. An intensity of 80% AMT was used. We applied the iTBS protocol in which 10 bursts of high frequency stimulation (3 pulses at 50 Hz) are delivered at 5 Hz every 10 s, for a total of 600 pulses (Huang et al., 2005). iTBS effects on both hemispheres were assessed by evaluating the changes of the RMT, AMT, and MEP amplitude stimulating the AH and UH, before and immediately after iTBS. MEP amplitude was evaluated as detailed above.

### Statistical analysis

Main aim of the statistical analysis is to assess the effect of gender on excitability and plasticity measures. Baseline and iTBS-dependent excitability changes were tested on RMT, AMT, MEP amplitude and on the Laterality Index (LI) (Cramer et al., 1997; Di Lazzaro et al., 2015). The latter is a derived compound estimate of inter-hemispheric excitability imbalance. In the case of MEP amplitude, LI is expressed by the following equation: LI_MEP_= (MEP_UH_- MEP_AH_)/(MEP_UH_ + MEP_AH_). On the contrary, in the case of AMT and RMT the correlation with excitability is opposite (the lower are the thresholds the higher is the excitability). Thus, LI is calculated as follow: LI_RMT_= (MEP_AH_ - MEP_UH_)/(MEP_UH_ + MEP_AH_) and LI_AMT_= (MEP_AH_ - MEP_UH_)/(MEP_UH_ + MEP_AH_). LI ranges between −1 and +1; positive values always indicate higher excitability of the UH. The bigger the difference from 0, the higher is the inter-hemispheric imbalance. Gender effect on baseline RMT, AMT, and MEP is evaluated applying a mixed model ANOVA with *Hemisphere* (two levels: *Affected -AH and Unaffected -UH*) as within-subjects factor and *Gender* (two levels: *Female and Male*) as between-subjects factor. A two-tailed independent sample t-test is used to assess the LI between groups difference. iTBS effect is tested on RMT, AMT, and MEP amplitude by using a mixed model ANOVA with *Hemisphere* (*Affected -AH and Unaffected -UH*) and iTBS (*Pre and Post*) as within-subjects factor and *Gender* (*Female and Male*) as between-subjects factor. The same model without the factor hemisphere is applied to study iTBS-related LI changes. Differences between females and males for non-normal distributed data were checked applying Mann-Whitney tests. In order to better address the variability of iTBS effects on MEP amplitude, the proportion of iTBS-induced AH excitability increase and UH excitability decrease are compared between groups, by means of Chi-Square test. The correlation between the clinical status and the neurophysiological data was performed using Pearson's correlation coefficients and partial correlations. The statistical distribution of all the variables is tested by means of Kolmogorov and Smirnov test. The significance level is set to 0.05. Descriptive statistic is reported as Mean ± Standard Error of the Mean (SEM).

## Results

The average NIHSS at onset was 5.21 ± 0.413. Gender groups were matched regarding age (F age = 69.87 ± 3.16; M age = 72.55 ± 2.50, *p* = 0.505) and clinical status (NIHSS) at stroke onset (F NIHSS = 4.73 ± 0.66; M NIHSS = 5.58 ± 0.53, *p* = 0.317). Groups were also matched regarding the percentage of patients with different lesion site (subcortical or cortical–subcortical), a pure cortical lesion was present in 3 out of 15 female patients (20%) and in 4 out of 20 male patients (20%). This is relevant because functional changes in cortical excitability may be influenced by stroke location and distribution (Ameli et al., 2009). Lesion size, as evaluated with the ASPECT score, was comparable in the two groups (*p* > 0.200) and resulted 7.47 ± 0.47 for females and 7.40 ± 0.36 for males. In a subgroup of 7 females and 8 males we measured the stroke volume using the procedure described in Di Lazzaro et al. (2010). The median stroke volume was 1463 mmc (range 653–26,514) for females and 2614 mmc (range 576–30,102) for males and it was not significantly different between males and females (Mann-Whitney *U*-test = 27.000, *p* = 0.955).

### Baseline brain excitability measures

**Figure 1:**
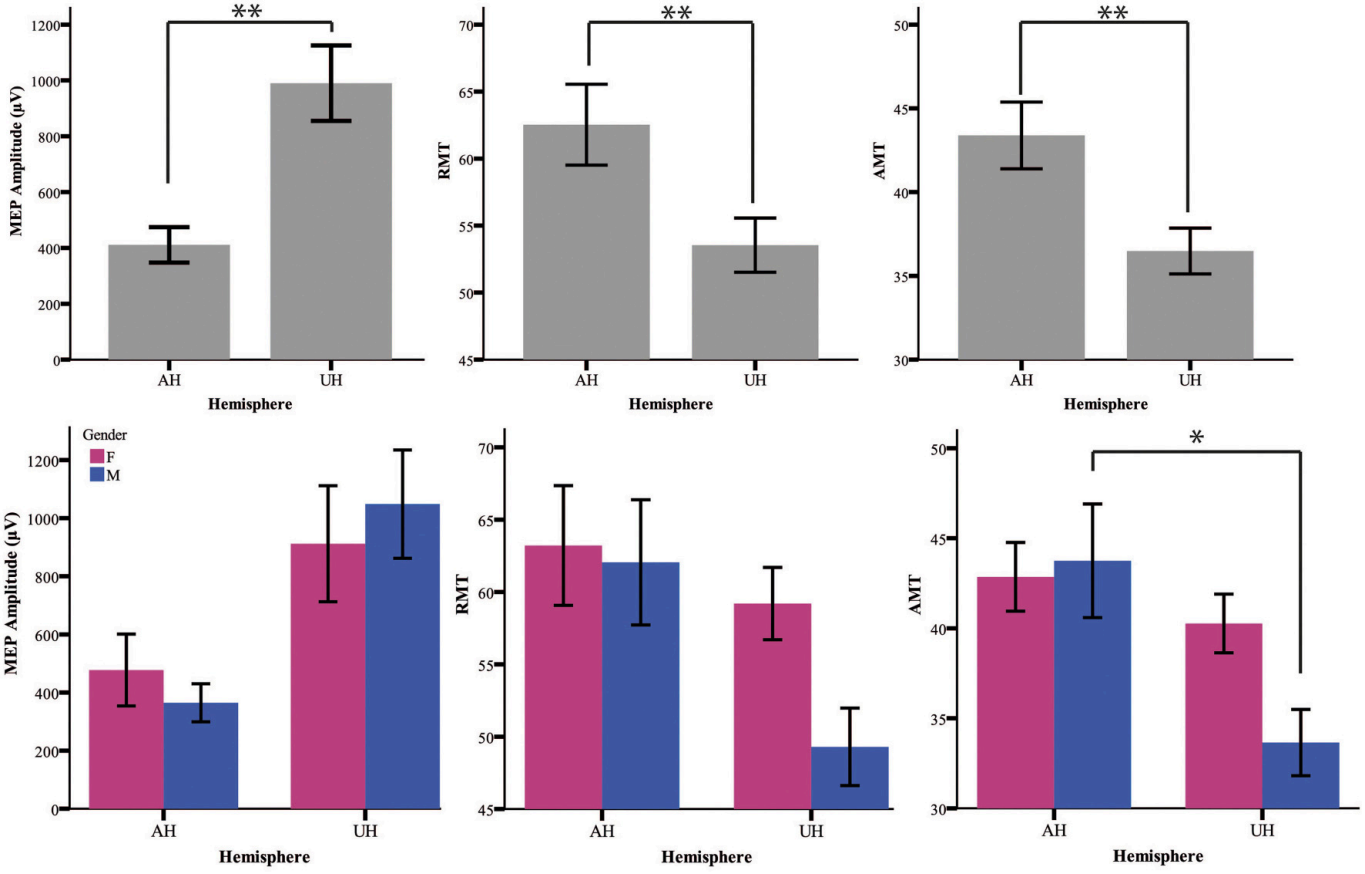
**Upper Panel:** Baseline excitability measures (MEP, RMT, AMT) in the different hemispheres pooling genders together. The statistical significance refers to the factor Hemisphere of the ANOVA model. **Lower Panel:** baseline value of excitability measures divided for gender (female = pink and male = blue). ^*^p < 0.05; ^**^p < 0.001 Error Bars = SER.

Table 1 summarizes the gender-related difference in basal and iTBS-induced changes. Considering all patients together, UH excitability is higher than AH excitability, as probed by RMT, AMT and MEP amplitude [Factor *Hemisphere*: *p* = 0.001, *p* = 0.001, *p* < 0.001, respectively. Figure 1 Upper Panel]. The effect of *Gender* on hemispheric excitability asymmetry, revealed by the *Hemisphere by Gender* interaction, is significant for AMT [*F*_(1, 32)_ = 4.449, *p* = 0.043], with a trend toward significance for RMT [*F*_(1, 32)_ = 3.412, *p* = 0.074], not significant for MEP amplitude [*F*_(1, 32)_ = 0.511, *p* = 0.480]. The *post-hoc* analysis reveals that AMT is significantly lower over the UH only in the *Male* group (*p* = 0.004), not in the *Female* group (*p* > 0.200) [Figure 1 Lower Panel and Tables 1, 2].

**Table 1:** **Summary of the gender-related difference in basal and iTBS-induced changes**.

**DIFFERENT POST-STROKE FUNCTIONAL CHANGES**	
On MTs	Males have higher AMT in the AH than in the UH (*p* = 0.004)
	Females have higher AMT in the UH than in the AH (*p* = 0.056)
On inter hemispheric balance	Males have higher inter-hemispheric asymmetry than females (LI_AMT_ *p* = 0.033 and LI_RMT_ *p* = 0.042)
	Males and Females have opposite inter-hemispheric balance (positive LI in males and negative in females)
**DIFFERENT PROPENSITY TO UNDERGO PLASTIC CHANGES**	
Pooling both hemispheres together	Females undergo a cumulative (pooling AH and UH together) increase of brain excitability, while males a decrease of it
Rate of iTBS effect	In the female group there is a higher rate of increase of AH excitability than in the male group (*p* = 0.022)

**Table 2:** **Excitability measures for both AH and UH under baseline conditions and after iTBS**.

		**RMT**						**AMT**						**MEP**					
		**PRE**			**POST**			**PRE**			**POST**			**PRE**			**POST**		
		**AH**	**UH**	**LI**	**AH**	**UH**	**LI**	**AH**	**UH**	**LI**	**AH**	**UH**	**LI**	**AH**	**UH**	**LI**	**AH**	**UH**	**LI**
F	Mean	63.21	59.20	−0.04	61.71	59.33	−0.06	42.86	40.27	−0.04	41.57	40.60	−0.06	477.42	911.95	0.40	634.75	792.27	0.29
	SER	4.14	2.50	0.07	4.35	2.49	0.07	1.91	1.63	0.07	1.94	1.63	0.07	123.64	199.45	0.10	170.69	160.87	0.12
M	Mean	62.05	49.30	0.10	62.05	50.70	0.09	43.75	33.65	0.12	42.90	34.70	0.09	364.51	1048.34	0.42	368.69	807.60	0.31
	SER	4.32	2.68	0.03	4.17	2.63	0.03	3.16	1.84	0.03	3.14	1.68	0.03	65.43	186.41	0.07	66.99	130.89	0.11
M+F	Mean	62.53	53.54	0.04	61.91	54.40	0.03	43.38	36.49	0.05	42.35	37.23	0.03	411.00	989.89	0.41	478.25	801.03	0.30
	SER	3.02	2.02	0.04	2.99	1.96	0.04	1.99	1.37	0.04	1.99	1.27	0.04	63.43	135.09	0.06	82.25	100.20	0.08

RMT, resting motor threshold, expressed as percentage intensity of maximal stimulator output; AMT, active motor threshold expressed as percentage intensity of stimulator output; MEP, amplitude of motor evoked potentials at 120% of RMT (μ V).

#### Laterality index

**Figure 2:**
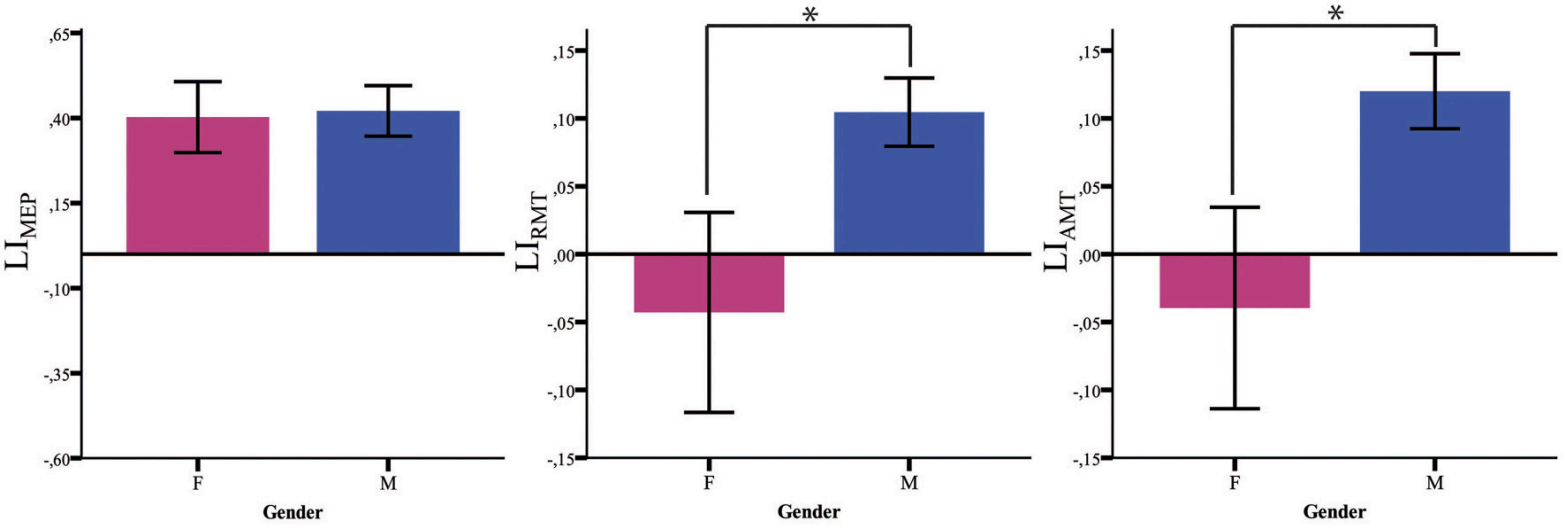
**Baseline LI measures**. LI baseline measures (MEP, RMT, AMT) in males (blue) and females (pink). ^*^p < 0.05; Error Bars = SER.

Both LI_AMT_ and LI_RMT_ show significant higher hemispheric asymmetry for the *Male* group (*p* = 0.042), while no significant difference has been found for LI_MEP_ (Figure 2 and Table 2).

### iTBS effects

**Figure 3:**
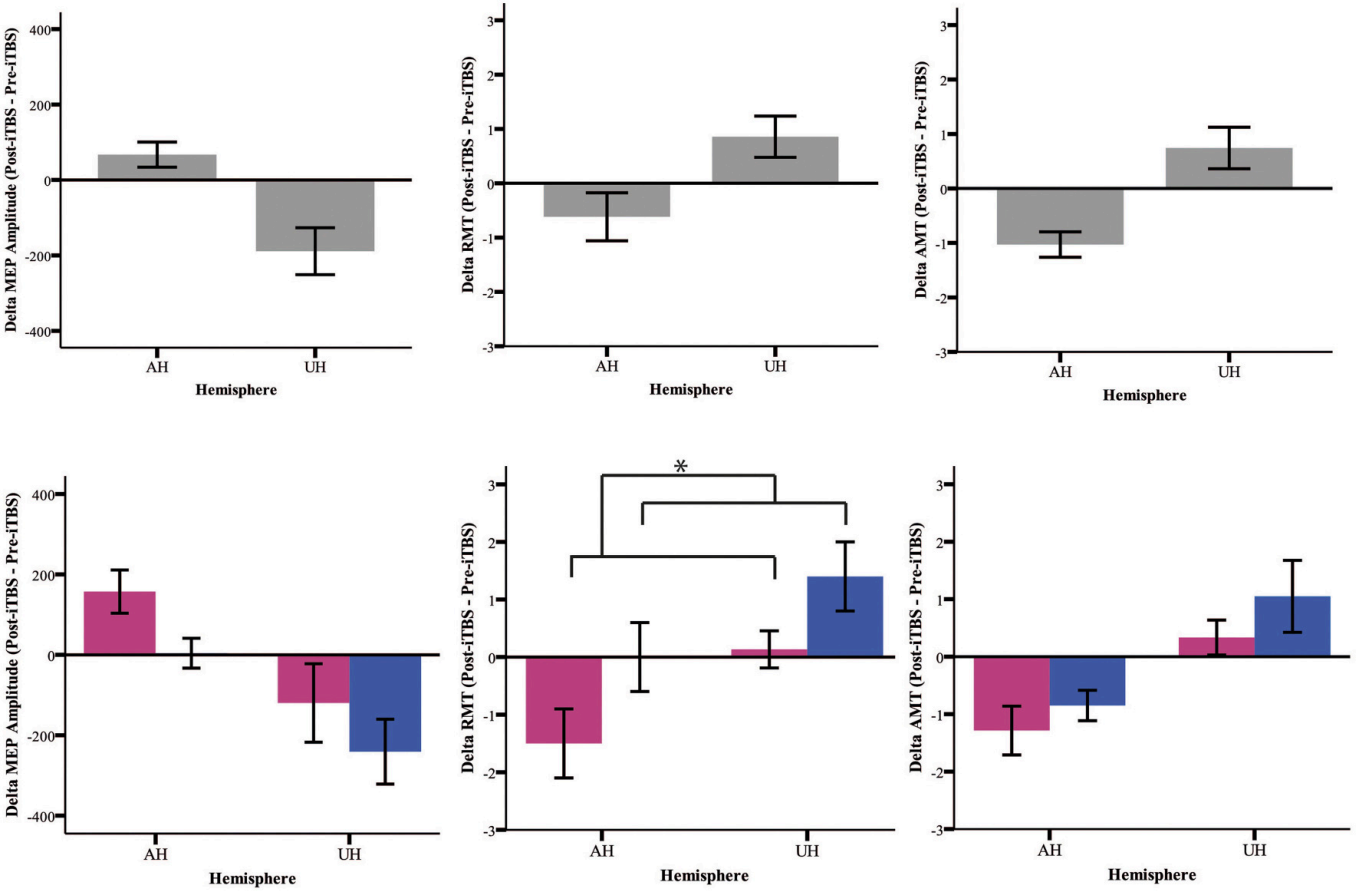
**Upper Panel: iTBS effects on excitability measures (MEP, RMT, AMT) on both groups together expressed as Post-iTBS—Pre-iTBS excitability change**. **Lower Panel:** iTBS effects on both hemispheres (AH and UH) and groups (female = pink and male = blue). iTBS effect on RMT is gender dependent [iTBS by Gender interaction *F*_(1, 32)_ = 7.860, ^*^p = 0.009].

Our analysis shows an *iTBS* by *Hemisphere* interaction [RMT *p* = 0.025; AMT *p* = 0.001; MEP *p* < 0.001], suggesting that there is an effect of iTBS characterized by an excitability increase over the AH and an excitability decrease over the UH (Figure 3, Tables 2, 3). RMT shows that *Gender* influences *iTBS* effects on brain excitability [*iTBS* by *Gender* interaction *F*_(1, 32)_ = 7.860, *p* = 0.009]. This effect does not differ depending on the *Hemisphere* [*iTBS* by *Gender* by *Hemisphere* interaction *F*_(1, 32)_ = 0.081, *p* = 0.777]. The significant *iTBS* by *Gender* interaction is motivated by a cumulative (both hemispheres together) mild increase of brain excitability for *Females* and decrease of brain excitability for *Males* (Figure 3, Tables 2, 3). *Gender* does not significantly impact on the effect of *iTBS* on AMT and MEP.

**Table 3:** **Summary of the ANOVA Mixed Model on iTBS effects on brain excitability measures**.

	**RMT**		**AMT**		**MEP**	
**Source**	***F*_(1, 32)_**	***p***	***F*_(1, 32)_**	***p***	***F*_(1, 32)_**	***p***
Hemisphere	9.462	0.004	8.779	0.006	12.778	0.001
iTBS	0.040	0.843	0.493	0.488	2.284	0.140
Gender	1.192	0.283	0.854	0.362	0.238	0.629
Hemisphere ^*^ iTBS	5.558	0.025	13.761	0.001	17.568	0.000
Hemisphere ^*^ Gender	3.465	0.072	4.316	0.046	0.834	0.368
iTBS ^*^ Gender	7.860	0.009	1.395	0.246	2.575	0.118
Hemisphere ^*^ iTBS ^*^ Gender	0.081	0.777	0.013	0.909	0.226	0.638

#### Laterality index

*iTBS* reduces the LI (LI_RMT_, LI_AMT_, LI_MEP_) regardless of the *Gender* [*iTBS* by *Gender* interaction: LI_RMT_ *F*_(1, 33)_ = 0.030, *p* = 0.864; LI_AMT_ *F*_(1, 33)_ = 0.223, *p* = 0.640; LI_MEP_ *F*_(1, 33)_ = 0.001, *p* = 0.979]. More in details:

- LI_RMT:_ *Pre-iTBS* 0.04 ± 0.04; *Post-iTBS* 0.03 ± 0.04, *p* = 0.022;
- LI_AMT:_*Pre-iTBS* 0.05 ± 0.04; *Post-iTBS* 0.03 ± 0.04, *p* = 0.001; after *iTBS* LI_AMT_ remains negative for *Female* and positive for *Male* [factor *Gender*: *F*_(1, 33)_ = 4.842, *p* = 0.035; *Post-iTBS* LI_AMT_ *Male* = 0.092 ± 0.028; *Female* = −0.060 ± 00.4];
- LI_MEP_: *Pre-iTBS* 0.41 ± 0.06; *Post-iTBS* 0.30 ± 0.08; *p* = 0.014.

To better characterize iTBS effects, we also looked at the rate of subjects reporting iTBS-related effects in the two groups. The rate of iTBS-induced AH excitability increase is higher in *Females* (14 out of 15, 93%) than in *Males* (11 out of 20, 55%; Chi-square *p* = 0.022). Even if the comparison does not reach a significant level (Chi-square *p* = 0.266), the rate of iTBS-induced UH excitability decrease is higher in *Males* (16 out of 20, 80%) than in *Females* (9 out of 15 female, 60%).

The individual level of brain excitability and iTBS effects are reported in Supplementary Figure 1.

### Relationship between clinical condition and neurophysiological measures

Pooling together all patients, NIHSS showed a significant correlation with the AMT LI both before iTBS (AMT LI pre-iTBS Pearson's *R* = 0.328, *p* = 0.029) and after iTBS (AMT LI post-iTBS Person's *R* = 0.327, *p* = 0.030). This correlation pattern did not survive the correction by sex, suggesting that sex might in fact play also a role in the relationship between measure of cortical excitability and clinical status. The subsequent analysis performed independently on the two groups showed: (i) absence of correlation in the male group (ii) strong correlation in the female group. More in details, in this subgroup we confirmed the relationship between LI and NIHSS (AMT LI Pre-iTBS Pearson's *R* = 0.500, *p* = 0.029, AMT LI Post-iTBS Pearson's *R* = 0.530, *p* = 0.021). In other words, a worst clinical condition is associated to stronger interhemispheric unbalance toward higher excitability of the UH. Additionally, both before and after iTBS higher NIHSS scores were associated to higher AH AMT (Person's *R* = 0.601, *p* = 0.011 before iTBS; Person's *R* = 0.649, *p* = 0.006, after iTBS).

## Discussion

Several studies have reported an asymmetry in the excitability of the AH and UH to non-invasive brain stimulation after stroke (Liepert et al., 2000; Manganotti et al., 2002; Shimizu et al., 2002; Cicinelli et al., 2003; Di Lazzaro et al., 2010, 2014). This is the first study evaluating the effects of gender on the changes in human brain excitability observed in the acute phase of stroke. We found sex differences in the functional changes that take place in AH and UH. The AH showed a lower excitability than the UH in both men and women, but males have higher excitability in the UH (lower AMT) and higher inter-hemispheric asymmetry than females. At a group level, the excitability of the AH is always lower than of the UH. However, the study of the LI, which takes into consideration and normalizes subject by subject for the level of excitability of both hemispheres, reveals that males and females have opposite inter-hemispheric balance, with higher excitability of UH in males, *vice versa* in females.

The meaning of these findings is still uncertain; we can speculate that they might be correlated with the existence of gender-related differences in the organizational patterns of functional cortical connectivity between different brain areas. Several studies have demonstrated sex differences in the connectivity of the brain (Gong et al., 2011). The results of the analysis of the structural connectome of the human brain suggest that male brains are structured to facilitate intra-hemispheric cortical connectivity, while female brain displays higher strength of inter-hemispheric connectivity (Ingalhalikar et al., 2014). Thus, we can speculate that to facilitate within-hemisphere connectivity in males there is a higher level of inter-hemispheric inhibition and, in case of a mono-hemispheric brain lesion, a lower level of inter-hemispheric inhibition from AH to UH makes the UH hyper-excitable to transcranial stimulation.

When testing the effects of iTBS of the AH, a rTMS protocol capable of inducing LTP-like changes in the stimulated hemisphere, females undergo a cumulative (pooling AH and UH together) increase of brain excitability, while males a decrease of it. In the female group, there is a higher rate of increase of AH excitability than in the male group and a tendency for a lower rate of decrease of UH excitability. Again men showed a pronounced effect in UH with a partial correction of the hyper-excitability associated with a comparable increase in the excitability of AH. In women, instead, we observed a more pronounced increase in the excitability of the AH that was associated with a slight increase in excitability, and not a suppression, of UH.

This is a further demonstration that the establishment of inter-hemispheric imbalance after stroke should not be given for grant, rather it is strictly dependent on patient's individuality. We recently showed that also the haplotype of Brain-Derived Neurotrophic Factor (BDNF) gene has a profound influence on the inter-hemispheric imbalance in cortical excitability (Di Lazzaro et al., 2015). Indeed, the presence of the Val66Met BDNF polymorphism is associated with a nine-fold weaker inter-hemispheric imbalance in cortical excitability as evaluated by comparing the RMT of the AH and the UH.

Is the imbalance in cortical excitability deleterious for recovery? As we suggested for BDNF polymorphism (Di Pino et al., 2014a, 2016), the hyper-excitability of UH might contribute to, or might interfere with, recovery depending on the level of impairment of AH. UH over-activity, observed more commonly in males, might interfere with paretic limb function in patients with less severe damage, while it might have a compensatory role in severely affected patients (Bradnam et al., 2013; Di Pino et al., 2014a). On the other hand, the limited imbalance between the hemispheres in females might represent an advantage in case of limited damage, facilitating the recovery of AH in the absence of a potentially deleterious interference from the UH. However, in more severe lesions the compensatory role of UH seems to be prevalent, and this might be limited in females by their lower UH hyper-excitability. Overall, this would reduce the impact of mild stroke in females and of more severe stroke in males, in line with a lower incidence, but a poorer prognosis of stroke in females and *vice versa* in males (Gibson, 2013).

Moreover, the differential functional changes that take place in the AH and UH in males and females might be adaptive or maladaptive depending on the degree of corticospinal tract damage. Gender influences many aspects of stroke including risk/incidence, diagnosis, symptoms, treatment and outcomes (Reeves et al., 2008; Appelros et al., 2009; Haast et al., 2012; Gibson, 2013); our study strongly contributes to highlight that it also influences the brain response to the damage.

Those considerations warrant further studies aimed at characterizing the interactions that gender and inter-hemispheric imbalance have on recovery.

In conclusion, our study suggests the existence of gender-dependent differences in the functional brain changes that take place after human stroke, in that it seems that male brain has greater asymmetry than the female's. This perfectly fits the recently advanced hypothesis of a higher strength of inter-hemispheric connection owned by the female's healthy brain (Ingalhalikar et al., 2014).

Male and female individuality could conceivably arise from a complex interaction of some sort of gender-specific base with a mosaic of environmental factors. Stroke and its strong plasticity-inducing potential are, in our opinion, optimal examples of events that might unveil and amplify those gender-specific differences, that otherwise might remain unrevealed. Our findings should suggest to be cautious in designing stroke studies, especially since sex differences in stroke that might affect recovery and brain plasticity probably result from a combination of factors, including elements intrinsic to the sex chromosomes, as well as the effects of sex hormone exposure, and not less important cultural and social factors (Cox et al., 2006; Vagnerova et al., 2008; Cesaroni et al., 2009; Liu et al., 2009; Gibson, 2013). For instance, animal model are often used to provide a better understanding of stroke and of specific brain recovery patterns (Alkayed et al., 1998; Bacigaluppi et al., 2010). However, the majority of experimental stroke studies keeps focusing on using only male animals (Fisher et al., 2009; Gibson, 2013), despite the Stroke Therapy Academy Industry Roundtable (STAIR) recommends that neuro-protective studies should be performed in both male and female rodents (Fisher et al., 2009). Moreover, the impacts of gender on the weight of age and hormone-related risk factors needs to be clarified, since epidemiological studies document an association between the female gender during the premenopausal years and a reduced risk of stroke addressing hormonal factors as potential protective treatments (Gibson et al., 2006, 2009; Suzuki et al., 2009; Liu and Yang, 2013). We envisage that a greater experimental plan and the understanding of the mechanisms underlying gender-related differences in stroke and responsiveness to neuroprotection and brain plasticity will lead to more appropriate treatment strategies for patients of both sexes.

## Author contributions

VD designed the study and wrote the manuscript. GP was in charge of statistic and revised the manuscript, GDP, FR, FL, LF, and FC participated to patients' recruitment, neuromodulation and revised the manuscript. All Authors approved the final version of the manuscript.

### Conflict of interest statement

The authors declare that the research was conducted in the absence of any commercial or financial relationships that could be construed as a potential conflict of interest.

## Supplementary material

Supplementary Figure 1 **Scatterplot graph of individual brain excitability levels (Post-iTBS over Pre-iTBS) for RTM, AMT and MEP Amplitude for the Affected (AH) and Unaffected (UH) hemispheres**.

## References

[B1] Alkayed N. J. Harukuni I. Kimes A. S. London E. D. Traystman R. J. Hurn P. D. (1998). Gender-linked brain injury in experimental stroke. Stroke 29, 159–165. Discussion: 166. 10.1161/01.STR.29.1.159 9445346

[B2] Ameli M. Grefkes C. Kemper F. Riegg F. P. Rehme A. K. Karbe H.. (2009). Differential effects of high−frequency repetitive transcranial magnetic stimulation over ipsilesional primary motor cortex in cortical and subcortical middle cerebral artery stroke. Ann. Neurol. 66, 298–309. 10.1002/ana.21725 19798637

[B3] Appelros P. Stegmayr B. Terént A. (2009). Sex differences in stroke epidemiology: a systematic review. Stroke 40, 1082–1090. 10.1161/STROKEAHA.108.540781 19211488

[B4] Bacigaluppi M. Comi G. Hermann D. M. (2010). Animal models of ischemic stroke. Part one: modeling risk factors. Open Neurol. J. 4, 26–33. 10.2174/1874205x01004010026 20802809 PMC2928914

[B5] Barber P. A. Demchuk A. M. Zhang J. Buchan A. M. (2000). Validity and reliability of a quantitative computed tomography score in predicting outcome of hyperacute stroke before thrombolytic therapy. Lancet 355, 1670–1674. 10.1016/S0140-6736(00)02237-6 10905241

[B6] Baron-Cohen S. Knickmeyer R. C. Belmonte M. K. (2005). Sex differences in the brain: implications for explaining autism. Science 310, 819–823. 10.1126/science.1115455 16272115

[B7] Bradnam L. V. Stinear C. M. Byblow W. D. (2013). Ipsilateral motor pathways after stroke: implications for non-invasive brain stimulation. Front. Hum. Neurosci. 7:184. 10.3389/fnhum.2013.00184 23658541 PMC3647244

[B8] Cesaroni G. Agabiti N. Forastiere F. Perucci C. A. (2009). Socioeconomic differences in stroke incidence and prognosis under a universal healthcare system. Stroke 40, 2812–2819. 10.1161/STROKEAHA.108.542944 19478229

[B9] Cicinelli P. Pasqualetti P. Zaccagnini M. Traversa R. Oliveri M. Rossini P. M. (2003). interhemispheric asymmetries of motor cortex excitability in the postacute stroke stage a paired-pulse transcranial magnetic stimulation study. Stroke 34, 2653–2658. 10.1161/01.STR.0000092122.96722.72 14551397

[B10] Cox A. M. Mckevitt C. Rudd A. G. Wolfe C. D. (2006). Socioeconomic status and stroke. Lancet Neurol. 5, 181–188. 10.1016/S1474-4422(06)70351-9 16426994

[B11] Cramer S. C. Nelles G. Benson R. R. Kaplan J. D. Parker R. A. Kwong K. K.. (1997). A functional MRI study of subjects recovered from hemiparetic stroke. Stroke 28, 2518–2527. 10.1161/01.STR.28.12.2518 9412643

[B12] Di Lazzaro V. Dileone M. Capone F. Pellegrino G. Ranieri F. Musumeci G.. (2014). Immediate and late modulation of interhemipheric imbalance with bilateral transcranial direct current stimulation in acute stroke. Brain Stimul. 7, 841–848. 10.1016/j.brs.2014.10.001 25458712

[B13] Di Lazzaro V. Pellegrino G. Di Pino G. Corbetto M. Ranieri F. Brunelli N.. (2015). Val66Met BDNF gene polymorphism influences human motor cortex plasticity in acute stroke. Brain Stimul. 8, 92–96. 10.1016/j.brs.2014.08.006 25241287 PMC4813754

[B14] Di Lazzaro V. Pilato F. Dileone M. Profice P. Oliviero A. Mazzone P.. (2008). The physiological basis of the effects of intermittent theta burst stimulation of the human motor cortex. J. Physiol. 586, 3871–3879. 10.1113/jphysiol.2008.152736 18566003 PMC2538925

[B15] Di Lazzaro V. Profice P. Pilato F. Capone F. Ranieri F. Pasqualetti P.. (2010). Motor cortex plasticity predicts recovery in acute stroke. Cereb. Cortex 20, 1523–1528. 10.1093/cercor/bhp216 19805417

[B16] Di Pino G. Pellegrino G. Assenza G. Capone F. Ferreri F. Formica D.. (2014a). Modulation of brain plasticity in stroke: a novel model for neurorehabilitation. Nat. Rev. Neurol. 10, 597–608. 10.1038/nrneurol.2014.162 25201238

[B17] Di Pino G. Pellegrino G. Capone F. Assenza G. Florio L. Falato E.. (2016). Val66Met BDNF polymorphism implies a different way to recover from stroke rather than a worse overall recoverability. Neurorehabil. Neural Repair. 30, 3–8. 10.1177/1545968315583721 25896987

[B18] Di Pino G. Pellegrino G. Capone F. Di Lazzaro V. (2014b). Human cerebral cortex metaplasticity and stroke recovery. Austin J. Cerebrovasc. Dis. Stroke 1:1001.

[B19] Draca S. (2010). Gender-specific functional cerebral asymmetries and unilateral cerebral lesion sequelae. Rev. Neurosci. 21, 421–425. 10.1515/REVNEURO.2010.21.6.421 21438191

[B20] Fisher M. Feuerstein G. Howells D. W. Hurn P. D. Kent T. A. Savitz S. I.. (2009). Update of the stroke therapy academic industry roundtable preclinical recommendations. Stroke 40, 2244–2250. 10.1161/STROKEAHA.108.541128 19246690 PMC2888275

[B21] Gibson C. L. (2013). Cerebral ischemic stroke: is gender important? J. Cereb. Blood Flow Metab. 33, 1355–1361. 10.1038/jcbfm.2013.102 23756694 PMC3764377

[B22] Gibson C. L. Coomber B. Rathbone J. (2009). Is progesterone a candidate neuroprotective factor for treatment following ischemic stroke? Neuroscientist 15, 324–332. 10.1177/1073858409333069 19359672

[B23] Gibson C. L. Gray L. J. Murphy S. P. Bath P. M. (2006). Estrogens and experimental ischemic stroke: a systematic review. J. Cereb. Blood Flow Metabolism 26, 1103–1113. 10.1038/sj.jcbfm.9600270 16437060

[B24] Gong G. He Y. Evans A. C. (2011). Brain connectivity: gender makes a difference. Neuroscientist 17, 575–591. 10.1177/1073858410386492 21527724

[B25] Haast R. A. Gustafson D. R. Kiliaan A. J. (2012). Sex differences in stroke. J. Cereb. Blood Flow Metabolism 32, 2100–2107. 10.1038/jcbfm.2012.141 23032484 PMC3519418

[B26] Huang Y. Z. Edwards M. J. Rounis E. Bhatia K. P. Rothwell J. C. (2005). Theta burst stimulation of the human motor cortex. Neuron 45, 201–206. 10.1016/j.neuron.2004.12.033 15664172

[B27] Ingalhalikar M. Smith A. Parker D. Satterthwaite T. D. Elliott M. A. Ruparel K.. (2014). Sex differences in the structural connectome of the human brain. Proc. Natl. Acad. Sci. U.S.A. 111, 823–828. 10.1073/pnas.1316909110 24297904 PMC3896179

[B28] Liepert J. Hamzei F. Weiller C. (2000). Motor cortex disinhibition of the unaffected hemisphere after acute stroke. Muscle Nerve 23, 1761–1763. 10.1002/1097-4598(200011)23:11<1761::AID-MUS14>3.0.CO;2-M 11054757

[B29] Lisabeth L. D. Reeves M. J. Baek J. Skolarus L. E. Brown D. L. Zahuranec D. B.. (2015). Factors influencing sex differences in poststroke functional outcome. Stroke 46, 860–863. 10.1161/STROKEAHA.114.007985 25633999 PMC4342324

[B30] Liu M. Dziennis S. Hurn P. D. Alkayed N. J. (2009). Mechanisms of gender-linked ischemic brain injury. Restor. Neurol. Neurosci. 27, 163–179. 10.3233/RNN-2009-0467 19531872 PMC2826890

[B31] Liu R. Yang S. H. (2013). Window of opportunity: estrogen as a treatment for ischemic stroke. Brain Res. 1514, 83–90. 10.1016/j.brainres.2013.01.023 23340160 PMC3664650

[B32] Manganotti P. Patuzzo S. Cortese F. Palermo A. Smania N. Fiaschi A. (2002). Motor disinhibition in affected and unaffected hemisphere in the early period of recovery after stroke. Clin. Neurophysiol. 113, 936–943. 10.1016/S1388-2457(02)00062-7 12048054

[B33] Narr K. Thompson P. Sharma T. Moussai J. Zoumalan C. Rayman J.. (2001). Three-dimensional mapping of gyral shape and cortical surface asymmetries in schizophrenia: gender effects. Am. J. Psychiatry 158, 244–255. 10.1176/appi.ajp.158.2.244 11156807 PMC2664826

[B34] Proust-Lima C. Amieva H. Letenneur L. Orgogozo J. M. Jacqmin-Gadda H. Dartigues J. F. (2008). Gender and education impact on brain aging: a general cognitive factor approach. Psychol. Aging 23, 608–620. 10.1037/a0012838 18808250

[B35] Reeves M. J. Bushnell C. D. Howard G. Gargano J. W. Duncan P. W. Lynch G.. (2008). Sex differences in stroke: epidemiology, clinical presentation, medical care, and outcomes. Lancet Neurol. 7, 915–926. 10.1016/S1474-4422(08)70193-5 18722812 PMC2665267

[B36] Rossini P. M. (2014). 1994-2014 Twenty years from the first guidelines for electrical and magnetic stimulation of brain, spinal cord and spinal roots. Clin. Neurophysiol. 125, 865–866. 10.1016/j.clinph.2014.01.004 24507859

[B37] Rossini P. M. Johnston C. S. (2005). Facilitating acute stroke recovery with magnetic fields? Neurology 65, 353–354. 10.1212/01.wnl.0000173428.48059.c7 16087896

[B38] Shimizu T. Hosaki A. Hino T. Sato M. Komori T. Hirai S.. (2002). Motor cortical disinhibition in the unaffected hemisphere after unilateral cortical stroke. Brain 125, 1896–1907. 10.1093/brain/awf183 12135979

[B39] Stefan K. Gentner R. Zeller D. Dang S. Classen J. (2008). Theta-burst stimulation: remote physiological and local behavioral after-effects. Neuroimage 40, 265–274. 10.1016/j.neuroimage.2007.11.037 18226550

[B40] Suppa A. Ortu E. Zafar N. Deriu F. Paulus W. Berardelli A.. (2008). Theta burst stimulation induces after−effects on contralateral primary motor cortex excitability in humans. J. Physiol. 586, 4489–4500. 10.1113/jphysiol.2008.156596 18669534 PMC2614023

[B41] Suzuki S. Brown C. M. Wise P. M. (2009). Neuroprotective effects of estrogens following ischemic stroke. Front. Neuroendocrinol. 30, 201–211. 10.1016/j.yfrne.2009.04.007 19401209 PMC3672220

[B42] Tomasi D. Volkow N. D. (2012). Laterality patterns of brain functional connectivity: gender effects. Cereb. Cortex 22, 1455–1462. 10.1093/cercor/bhr230 21878483 PMC3450858

[B43] Vagnerova K. Koerner I. P. Hurn P. D. (2008). Gender and the injured brain. Anesth. Analg. 107, 201–214. 10.1213/ane.0b013e31817326a5 18635489 PMC2651745

