# Supplementary figures and images for: Human Motor Cortex Functional Changes in Acute Stroke: Gender Effects
Source: Front Neurosci. 2016 Jan 29;10:10. doi: 10.3389/fnins.2016.00010 (PMC4731507; doi:10.3389/fnins.2016.00010)

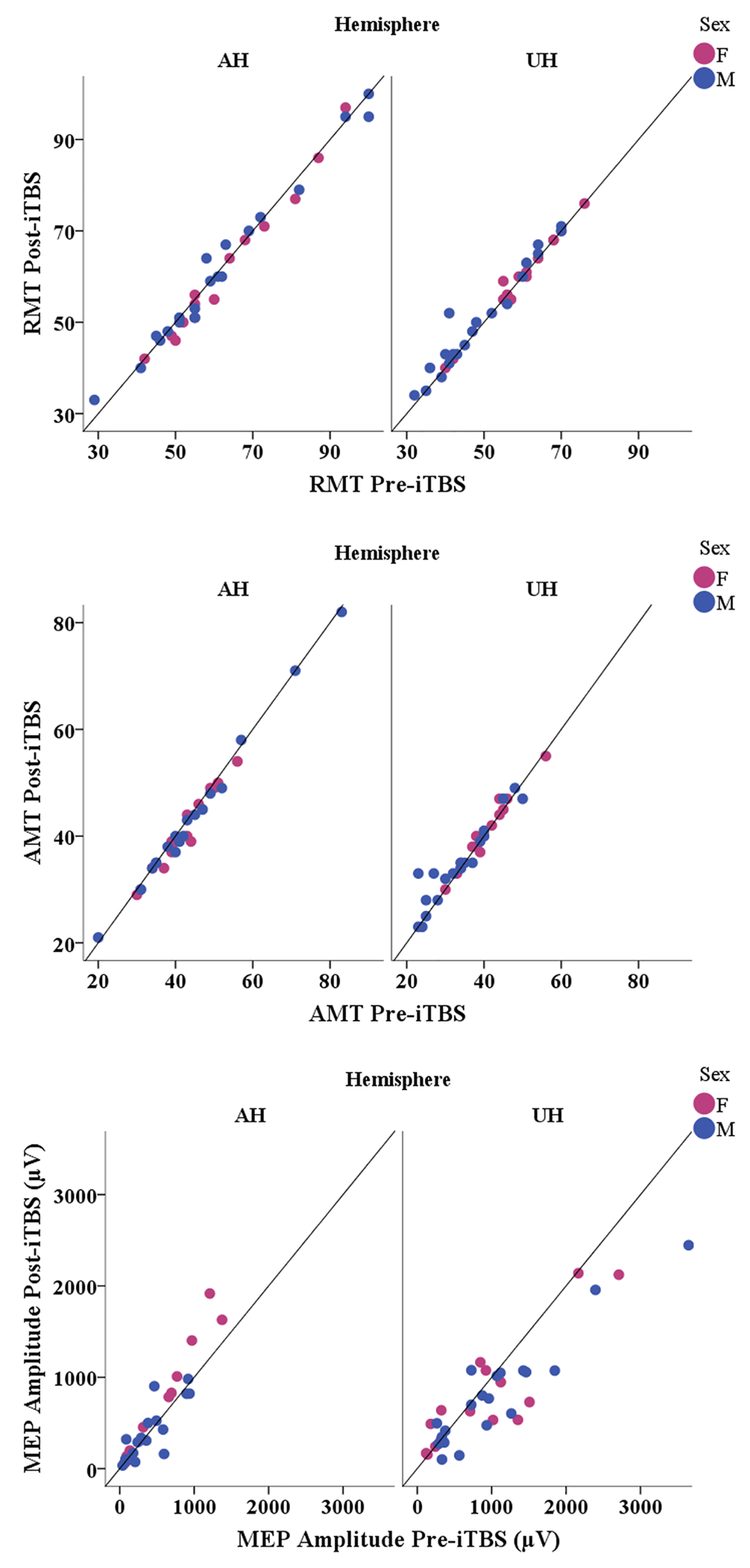

Supplement: Supplementary Figure 1 — Scatterplot graph of individual brain excitability levels (Post-iTBS over Pre-iTBS) for RTM, AMT and MEP Amplitude for the Affected (AH) and Unaffected (UH) hemispheres. [file Image1.tif]
